# Chlorine Inactivation of Highly Pathogenic Avian Influenza Virus (H5N1)

**DOI:** 10.3201/eid1310.070323

**Published:** 2007-10

**Authors:** Eugene W. Rice, Noreen J. Adcock, Mano Sivaganesan, Justin D. Brown, David E. Stallknecht, David E. Swayne

**Affiliations:** * US Environmental Protection Agency, Cincinnati, Ohio, USA; † University of Georgia, Athens, Georgia, USA; ‡ US Department of Agriculture, Athens, Georgia, USA

**Keywords:** Highly pathogenic avian influenza, H5N1, chlorination, water, dispatch

## Abstract

To determine resistance of highly pathogenic avian influenza (H5N1) virus to chlorination, we exposed allantoic fluid containing 2 virus strains to chlorinated buffer at pH 7 and 8, at 5°C. Free chlorine concentrations typically used in drinking water treatment are sufficient to inactivate the virus by >3 orders of magnitude.

Growing concerns about the public health threat posed by highly pathogenic avian influenza (HPAI) subtype H5N1 has prompted interest in evaluating environmental control measures for this virus. The World Health Organization has noted that more information is needed on the effectiveness of inactivation of subtype H5N1 in water (*1*). Since 2002, HPAI (H5N1) has been reportedly isolated from >50 different wild avian species, mainly aquatic birds in the order Anseriformes (*2*). Experimentally infected waterfowl shed moderate to large quantities of the virus in their feces and respiratory secretions (*3*,*4*). HPAI viruses can persist in simulated water environments, although generally for shorter periods than low pathogenic avian influenza viruses (*5*,*6*). Open bodies of water, including drinking water reservoirs, can become contaminated by birds that are actively shedding virus or by waterfowl carcasses. Surface runoff also represents a potential source of contamination for groundwater. In terms of avian health, drinking water has been implicated in the transmission of avian influenza among domestic poultry (*6*–*8*).

Chlorination represents the most common form of disinfection used in water treatment. Most published reports on virus inactivation in water have dealt with enteric viruses, and government guidelines for water treatment have focused on this group. Despite general acceptance that the outer lipid envelope associated with influenza viruses would make them susceptible to chlorination, no published reports specifically address the effect of chlorine on the H5N1 subtype of avian influenza.

## The Study

Two clade 2 strains of HPAI (H5N1) virus were used in this study (*9*): 1 isolated from domestic poultry, A/chicken/Hong Kong/D-0947/2006 (courtesy of K. Dytring; Agriculture, Fisheries and Conservation Department, Hong Kong Special Administrative Region of China) (*10*), and 1 from a wild swan, A/WhooperSwan/Mongolia /244/2005 (*3*). The infectious virus was propagated in embryonated eggs of specific pathogen-free (SPF) leghorn chickens (*11*), and infective amnioallantoic fluid was harvested 96 h after inoculation.

Inactivation experiments were conducted as previously described (*12*). The initial chlorine level was chosen to achieve a chlorine residual that would be typical of drinking water after satisfying the initial chlorine demand of the amnioallantoic fluid. Briefly, virus-infected allantoic fluid was diluted (1:1,000) into continuously stirred, chlorinated, chlorine demand–free phosphate buffer (0.05 M, pH 7.0 and 8.0). Chlorine measurements were made immediately before the chlorine was neutralized by the addition of 0.1 mL of sodium thiosulfate (10% w/v). Separate reaction vessels were used for each exposure time. Reaction vessels containing only the virus and buffer without chlorine served as controls for determination of virus titers in the absence of chlorine and were assayed at the end of the longest exposure time period (60 s). Negative buffer controls without virus or chlorine were also included. All test and control samples were treated in the same manner. Preliminary investigations indicated that the virus can be readily inactivated at room temperature (data not shown). To slow the rate of inactivation, experiments were conducted at 5°C.

The infectivity of the samples was quantified by using microtiter endpoint titration (*6*)*,* and virus titers were expressed as median 50% tissue culture infectious dose (TCID_50_)/mL (*13*). Primary cultures of chicken embryo fibroblasts prepared from 9- to 11-day-old SPF chicken embryos were used in these assays. Virus-infected cells were incubated at 37^o^C under 5% CO_2_ for 96 h and examined by light microscopy for cytopathic effect (CPE). Culture plates were stained with 1% (w/v) crystal violet in 10% (v/v) neutral-buffered formalin for further examination. Failure to produce CPE indicated that the virus was not capable of infecting the cells. The neutralized buffer control without virus did not cause CPE. All experiments were conducted in duplicate under Biosafety Level 3 agricultural conditions.

Inactivation levels were determined by comparing the log_10_ transformed TCID_50_/mL virus titers in the control samples with the titers in the chlorine-exposed samples. The lowest detectable virus titer was 2.17 log_10_ TCID_50_/mL, independent of pH or virus strain. *Ct* values (the chlorine concentration, *C* [mg/L], multiplied by the exposure time, *t* [min]) were used to determine the rate of inactivation for the 2 pH levels. *Ct* values are commonly used to make disinfection recommendations for water treatment and provide a means for comparing biocidal activity for various microorganisms (*14*)*.* *Ct* values were plotted against log_10_ virus titers to determine *Ct* values for a given level of inactivation (Table 1).

**Table 1:** *Ct* values (mg-min/L) for inactivation of HPAI (H5N1) virus by free chlorine at 5ºC*

Strain	pH	Log_10_ inactivation		
1.0	2.0	3.0
Hong Kong†	7	0.14	0.27	0.41
	8	0.26	0.53	0.79
Mongolia‡	7	0.13	0.26	0.39
	8	0.23	0.46	0.68

*Ct, 0.5(*C*_0_ + *C*_0.5_)/2 + *C*_0.5_ [1–exp (–k (*t*–0.5) ]/k, where *C*_0_ = chlorine concentration at time zero (mg/L); *C*_0.5,_ chlorine concentration at 0.5 min (mg/L); k, exponential chlorine decay rate; *t* = time (min). HPAI, highly pathogenic avian influenza. †A/chicken/Hong Kong/D-0947/2006. ‡A/WhooperSwan/Mongolia/244/2005.

The results of the chlorination experiments (Table 2) represent the means of duplicate experiments differing by <0.10 mg/L of free available chlorine. Initial titers of all virus preparations yielded log_10_TCID_50_/mL values >5.26, which enabled *Ct* calculations for inactivation over several orders of magnitude. The A/chicken/Hong Kong/D-0947/2006 strain preparations exhibited a slightly higher chlorine demand, ≈1.5 mg/L after 1 min, compared with 1.0 mg/L for the A/WhooperSwan/Mongolia/244/2005 strain during the same time interval. As anticipated, inactivation was slower at pH 8.0 than at pH 7.0. Table 1 lists the mean *Ct* values (mg-min/L) required to achieve 1, 2, and 3 orders of magnitude inactivation for both strains at the 2 pH levels. Covariance analysis of the decay coefficients indicated no significant difference in the inactivation of the 2 virus strains at pH 8.0 (p = 0.10). Rapid inactivation at pH 7.0 did not allow for statistical evaluation.

**Table 2:** Inactivation of HPAI (H5N1) virus by free chlorine at 5°C*

Strain	pH	Time, s	Free chlorine, mg/L	Virus titer log_10_ TCID_50_/mL	Log_10_ reduction
Hong Kong†	7	0	2.08	5.32	NA
		15	ND	<2.17	>3.15
		30	0.65	<2.17	>3.15
		60	0.52	<2.17	>3.15
	8	0	2.08	5.70	NA
		15	ND	3.88	1.82
		30	0.76	2.67	3.03
		60	0.59	<2.17	>3.53
Mongolia‡	7	0	1.86	5.26	NA
		15	ND	<2.17	>3.09
		30	0.85	<2.17	>3.09
		60	0.77	<2.17	>3.09
	8	0	2.04	5.53	NA
		15	ND	3.39	2.14
		30	1.10	<2.17	>3.36
		60	1.08	<2.17	>3.36

*HPAI, highly pathogenic avian influenza; TCID_50,_ median 50% tissue culture infectious dose; NA, not applicable; ND, not determined. †A/chicken/Hong Kong/D-0947/2006. ‡A/WhooperSwan/Mongolia/244/2005.

## Conclusions

The results of this study confirm that avian influenza (H5N1) is readily inactivated by chlorination. Although the viral inoculum exerted a considerable initial chlorine demand, the maintenance of a free chlorine residual (0.52–1.08 mg/L) was sufficient to inactivate the virus by >3 orders of magnitude within an exposure time of 1 minute. Chlorine demand would also be anticipated when the virus is associated with fecal material. These findings indicate that the ability to compensate for an initial chlorine demand followed by exposure to a relatively low level of free chlorine for a short time is sufficient to inactivate the virus by chlorination. For drinking water disinfection at conditions similar to those used in this study, the US Environmental Protection Agency specifies free chlorine *Ct* values of 6 and 8 mg-min/L to achieve enteric virus inactivation of 3 and 4 orders of magnitude, respectively (*14*). According to our results, these *Ct* values would be more than sufficient to inactivate HPAI (H5N1) in the water environment. The information on chlorine disinfection presented here should be helpful for developing risk management procedures regarding the role of water in the transmission of the virus to humans and poultry.

## References

[1] World Health Organization. Review of latest available evidence on risks to human health through potential transmission of avian influenza (H5N1) through water and sewage. WHO/SDE/WSH/06.1. Geneva: The Organization; 2006.

[2] National Wildlife Health Center. List of species affected by H5N1 (avian influenza). Nov 2006. [cited 2007 Jul 25.] Available from http://www.nwhc.usgs.gov/disease_information/avian_influenza/affected_species_chart.jsp</eref>

[3] Brown JD, Stallknecht DE, Beck JR, Suarez DL, Swayne DE. Susceptibility of North American ducks and gulls to H5N1 highly pathogenic avian influenza viruses. Emerg Infect Dis. 2006;12:1663–70. 17283615 10.3201/eid1211.060652 PMC3372354

[4] Sturm-Ramirez KM, Ellis T, Bousfield B, Bissett L, Dyrting K, Rehg JE, Reemerging H5N1 influenza viruses in Hong Kong in 2002 are highly pathogenic to ducks. J Virol. 2004;78:4892–901. 10.1128/JVI.78.9.4892-4901.2004 15078970 PMC387679

[5] Stallknecht DE, Shane SM, Kearney MT, Zwank PJ. Persistence of avian influenza viruses in water. Avian Dis. 1990;34:406–11. 10.2307/1591428 2142420

[6] Brown JD, Swayne DE, Cooper RJ, Burns RE, Stallknecht DE. Persistence of H5 and H7 avian influenza viruses in water. Avian Dis. 2007;51(Suppl 1):285–9. 10.1637/7636-042806R.1 17494568

[7] Sivanandan V, Halvorson DA, Laudert E, Senne DA, Kumar MC. Isolation of H13N2 influenza A virus from turkeys and surface water. Avian Dis. 1991;35:974–7. 10.2307/1591638 1838479

[8] Laudert E, Sivanandan V, Halvorson DA, Shaw D, Webster RG. Biological and molecular characterization of H13N2 influenza type A viruses isolated from turkeys and surface water. Avian Dis. 1993;37:793–9. 10.2307/1592031 8257373

[9] World Health Organization Global Influenza Program Surveillance Network. Evolution of H5N1 avian influenza viruses in Asia. Emerg Infect Dis. 2005;11:1515–21. 16318689 10.3201/eid1110.050644 PMC3366754

[10] Smith GJ, Fan XH, Wang J, Li KS, Qin K, Zhang JX, Emergence and predominance of H5N1 influenza variant in China. Proc Natl Acad Sci U S A. 2006;103:16936–41. 10.1073/pnas.0608157103 17075062 PMC1636557

[11] Swayne DE, Senne DA, Beard CW. Influenza. 4th ed. In: Swayne DE, Glisson JR, Jackwood MW, Pearson JE, Reed WM, editors. Kennett Square (PA): American Association of Avian Pathologists; 1998.

[12] Rice EW, Clark RM, Johnson CH. Chlorine inactivation of *Escherichia coli* O157:H7. Emerg Infect Dis. 1999;5:461–3. 10.3201/eid0503.990322 10341188 PMC2640762

[13] Reed LJ, Muench H. A simple method for estimating fifty per cent endpoints. Am J Hyg. 1938;27:493–7.

[14] United States Environmental Protection Agency. Guidance manual for compliance with the filtration and disinfection requirements for public water systems using surface water sources. 1991; Washington: The Agency; 1991.

